# Micro-targeting consumers to reduce consumptive externalities

**DOI:** 10.1371/journal.pone.0284338

**Published:** 2023-05-04

**Authors:** Tamara L. Sheldon, J. R. DeShazo, Bronwyn Lewis Friscia

**Affiliations:** 1 Department of Economics, Darla Moore School of Business, University of South Carolina, Columbia, SC, United States of America; 2 Lyndon B. Johnson School of Public Affairs, University of Texas at Austin, Austin, TX, United States of America; University of Naples Federico II: Universita degli Studi di Napoli Federico II, ITALY

## Abstract

When correcting for consumption externalities policymakers may employ economic incentives, a uniform moral suasion intervention, or various micro-targeted moral suasion interventions. To assess the relative effectiveness of these policy interventions, we randomly assign consumers to different moral suasion treatments designed to increase their willingness to pay for energy efficient light bulbs. Both economic incentives and single moral suasion interventions have similar modest effects on household willingness to pay for this durable good. However, we find that optimally targeting moral suasion messages increases consumers’ choice of the most efficient light bulbs even more than large subsidies.

## 1 Introduction

As efforts grow to internalize the externalities causing climate change and other forms of pollution, researchers have begun to assess the relative effectiveness of policy interventions that alter economic incentives versus those that use moral suasion [1, 2]. Interest in this topic has been motivated by recent findings that consumers imperfectly respond to changes in the marginal prices of many externality-causing commodities such as electricity [3, 4], natural gas [5], and water [6]. Purchases of externality-producing durable goods exhibit a similar phenomenon. For example, consumers appear to be imperfectly sensitive to the energy-related variable costs associated with housing [7], vehicles [8, 9] and appliances [10, 11]. Collectively, these findings have raised questions about the effectiveness of policy interventions motivated by the idea of Pigouvian taxes and prompted a search for alternative interventions. They have also raised questions about whether economic incentives and moral suasion as policy interventions should be viewed as substitutes or complements for one another.

In this paper, we provide a head-to-head test of the policy effectiveness of subsidies, homogenously-targeted externality messages, and micro-targeted externality messages for a durable good. Specifically, we assess a second-best subsidy that is provided only to consumers who purchase a “best-in-class” durable good that has either the highest relative efficiency rating or employs a cleaner technology. This is by far the most common type of subsidy used to incentivize the purchase of a very broad set of durable goods such as automobiles, household appliances, and building technologies related lighting, heating, cooling, and water service. The paper closest to ours is [2], which focuses on electricity demand to find that moral suasion has only short-term effects but can be withdrawn and then effectively re-introduced. The authors also find that price increases have more enduring effects on household consumption of energy. However, we focus on households’ episodic purchases of a durable good (e.g., light bulbs), rather than a commodity that is continuously consumed (e.g., electricity). We also test for the effects of lump-sum subsidies for the most energy efficient version of a good as compared to changing the marginal price of a commodity. Lastly, we evaluate the targeting of distinct moral messages that are micro-targeted to each consumer rather that assigning a uniform, general moral suasion message.

Because they are relatively low-cost durable goods, lightbulbs have been used to explore technology diffusion [12], policy welfare effects [13], social pressure from the experimenter [14], and political ideology [15]. While we cannot extrapolate our findings to more expensive durable goods, we are able to assess several innovative hypotheses with respect to the intensive margin of choice for this durable good. In doing so, our research also complements a growing literature focused on consumers’ extensive choice of carbon-intensive commodities that are durable good inputs. See [16] on evidence of nudges for larger durables goods. Other research suggests consumers may respond more strongly to financial messaging as the magnitude of the financial gains increase [17, 18].

Our study begins by estimating willingness to pay (WTP) for energy efficiency in the context of light bulbs using data gathered from a national online choice experiment with a field experiment component. Within this choice experiment we randomly assign two moral suasion treatments, an environmental message and an energy independence message, both of which highlight negative externalities associated with electricity consumption. A third randomly assigned message focuses on frugality by considering the “best value for the money.” We consider this the control because rather than highlight a negative externality, it focuses on the private benefits of consumption using a ubiquitous “value” framing. We administered our online survey and field experiment module to a nationally representative sample of 1,802 voting-age U.S. adults. Our research design combines stated and revealed preference data collected from six choice experiments, each involving a choice between two light bulbs with varying levels of price, lifespan, and energy efficiency. In the final choice experiment, respondents were faced with a real purchase decision. Consistent with earlier insights about the role of heterogeneity in correcting consumption externalities [19], we find significant variation in consumer responses to alternative moral suasion treatments that results in heterogeneous willingness to pay for energy efficiency. In experimental design, our study is most closely related to [20], who perform a field experiment in China, presenting participants with an efficient LED lightbulb and a less efficient incandescent lightbulb and using a multiple price list to reveal WTP for the LED. Treatments included combinations of 1) questions about expected lifetime costs, 2) questions about environmental effects, and 3) information about relative lifetime costs of the two types of bulbs. They conclude that the energy efficiency gap is not driven by biased beliefs or salience, but rather, uncertainty of long term cost savings from adopting the more efficient lightbulb.

The second component of our study involves simulating energy efficiency decisions under the assumption that the policy maker is able to optimally “micro-target” each consumer with the most effective of the three suasion messages. The only study we’re aware of that uses machine learning to predict household energy use response to policy is [21]. The author randomly assigned electricity pricing schemes and information treatments to households in Ireland, then used a machine learning model to estimate responses of household electricity use to time-of-use prices. He finds that consumer awareness, information provision, and baseline consumption are the most important sources of heterogeneity out of more than 150 household observables.

We show that this micro-targeting increases adoption of energy efficient lightbulbs significantly more than either a homogeneously-assigned messaging campaign or a substantial subsidy for the most energy efficient light bulbs, reducing energy consumption by up to 5.7% versus 4.6% or 1.8%, respectively. This is despite that fact that we assess a relatively large $1 subsidy for the purchase of the most efficient light bulbs, which represents a 29% price reduction of the average purchase price ($3.43) of selected bulbs in the choice experiment.

We find that this economic incentive does not perform quite as well as the uniformly-assigned moral suasion treatments. Targeting all consumers with the environmental message leads to a slightly larger increase in energy efficiency than does a $1 subsidy, reducing energy consumption by an additional 0.7%. Targeting all consumers with the energy independence message performs better, reducing energy consumption by an additional 2.8%. However, neither of these two types of policy interventions performs as well as ex ante micro-targeting the different moral suasion messages based on observable differences across consumers. We find that optimal micro-targeting leads to 22% of consumers receiving the environmental message, 36% the energy independence message, and 43% the frugality message. Finally, we assess whether micro-targeting the subsidies to only lower-income consumers changes our comparative results only to find that it does not.

Our moral suasion interventions provide information on previously-omitted product attributes that are relevant to specific types of externalities. Our intervention differs from other commonly studied non-financial interventions that rely on either social comparisons [22, 23] or social recognition [24]. These interventions, which provide information on the relative social performance of consumers, may induce utility increases when they socially frame the consumer positively and disutility when they socially frame them negatively [25]. Although our policy interventions provide no direct socially-comparative information, it is nevertheless possible that consumers might be willing to pay either to avoid or to receive the externality-focused attribute information that our treatments provide. Researchers have documented that people are often willing to pay to avoid the information or to signal their information preferences by avoiding the information. See, for example, [22, 26–29]. Since we do not evaluate these additional welfare impacts of our treatments, our analysis does not represent a comprehensive welfare assessment of our moral suasion policy strategies, but rather a more behaviorally-focused assessment of micro-targeting their impacts on consumer choice outcomes.

The paper proceeds as follows. After explaining the survey and data in Section 2 we present the empirical model in Section 3. In Section 4 we present the results of the empirical model estimation (Section 4.1), use simulations to investigate policy implications (Section 4.2), and explore micro-targeting (Section 4.3). Section 5 concludes.

## 2 Survey design and data

Our choice experiment is based on a nationally representative sample of voting-age U.S. adults in April 2018 and collected a sample of 1,802 completed surveys. We worked with the GfK Group, formerly Knowledge Networks, to conduct this study. The sample was drawn from GfK’s KnowledgePanel, which is a probability-based web panel designed to be nationally representative. IRB approval for this study was granted by the University of California, Los Angeles Institutional Review Board. Approval was granted 3/9/2018 and the record number is IRB#18- 000054. The UCLA IRB waived the requirement for signed informed consent for the research under 45 CFR 46.117(c)(2). Of the respondents who completed an initial screening question, roughly 48.5% both qualified as potential light bulb purchasers for their household and completed the survey in its entirety. S1 Table in S1 File contains a table comparing the socio-demographics of our sample to those of the U.S. population. At the outset of the survey, respondents were randomized into one of three survey types corresponding to our three treatments, which will be explained in detail below.

The survey opened by gathering data on the respondents’ political beliefs, values, and light bulb use. The survey introduced one at a time the three features of light bulbs—price, lifespan, and level of energy efficiency—that would vary in the choice sets that would later be presented to them, as well as their typical attribute ranges. These displays were shown on three successive screens to encourage reading comprehension and reduce text fatigue. Respondents were then asked to rank these attributes in order of their importance to them when purchasing light bulbs. Although the attributes were shown in the same order to all respondents, given the brief nature of these screens and the position towards the beginning of the survey, inattention is unlikely an issue. Additionally, roughly the same proportion of respondents indicated each of the three attributes as the most important to them following the attribute introduction: 32.8% selected price, 31.0% selected lifespan, and 36.2% energy efficiency. Furthermore, if inattention were an issue in the choice experiment, it would lead to a downward bias and larger standard errors for the estimated coefficients.

**Fig 1:**
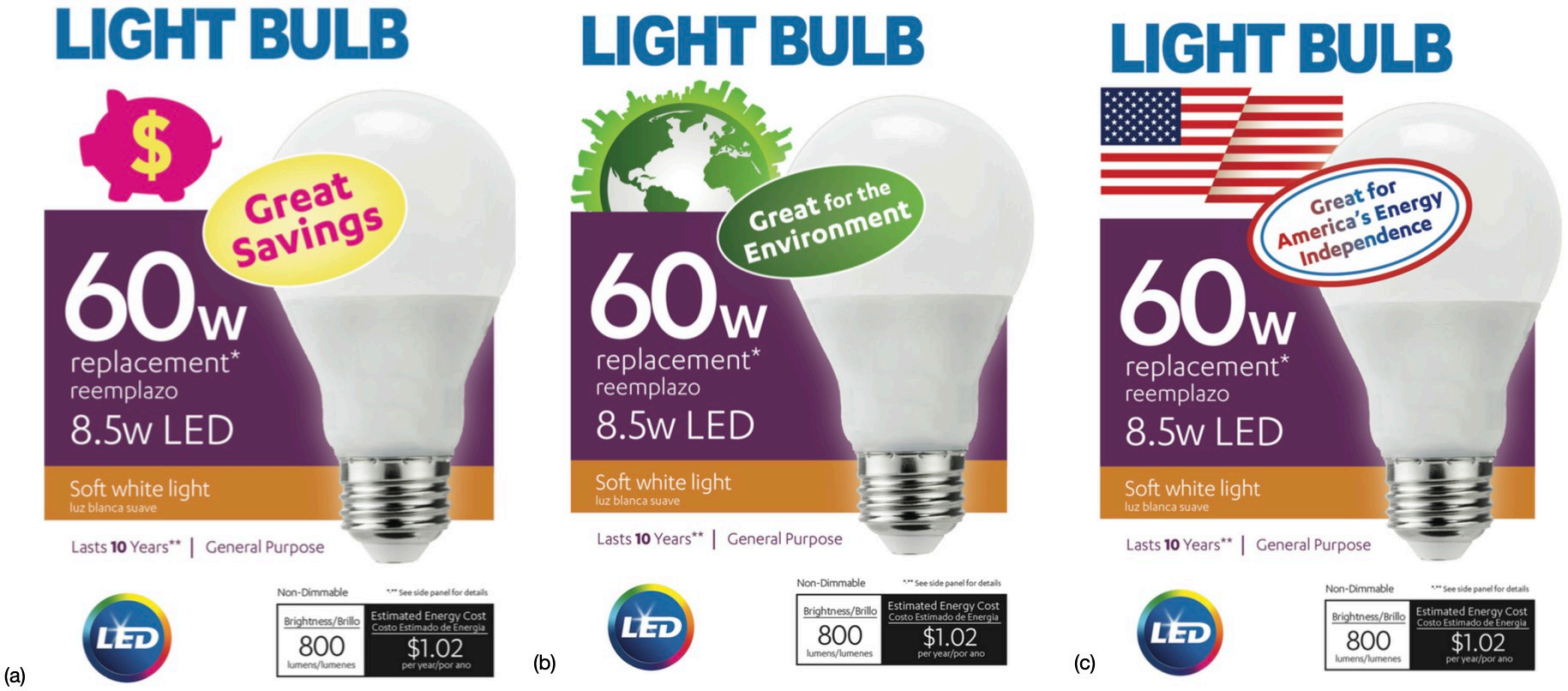
Messages for control and externality treatments. A: Frugality Treatment (Control). B: Environmental Treatment. C: Energy Independence Treatment.

Respondents were shown one of three treatments, each of which expressed a different message as to why a consumer might prioritize energy efficiency when choosing a light bulb to purchase. Each of the three explanations were accompanied by a visual prime displaying an image of light bulb packaging that marketed the product with the motivation for energy efficiency highlighted in the message, as shown in Fig 1. In the frugality treatment, our control condition, respondents were shown a message that simply emphasized the cost savings of the purchase. Respondents were told that energy efficient light bulbs “save you money by reducing how much you spend on electricity each year”; the visual prime displayed a pink piggy bank with a dollar symbol and the slogan “Great Savings.” The other two treatments, environmental and energy independence, highlight two negative externalities associated with electricity consumption- greenhouse gas emissions and energy dependence. In the environmental treatment, respondents were treated with a message informing them that energy efficient light bulbs “reduce the greenhouse gas emissions that cause climate change”; the visual prime displayed a green earth symbol and the slogan “Great for the Environment.” In the energy independence treatment, respondents were treated with a message informing them that energy efficient light bulbs “reduce America’s dependence on the foreign countries that we currently purchase oil and natural gas from”; the visual prime displayed red, white, and blue American flag imagery and the slogan “Great for America’s Energy Independence.” Table 1 shows summary statistics of the full sample as well as the subsamples according to randomized treatment.

**Table 1:** Summary statistics of respondents.

	Full Sample	Frugality	Environmental	Energy Indep.
Conservative	37.7%	37.3%	39.5%	36.4%
Liberal	35.1%	36.8%	32.5%	35.9%
Neither Conservative nor Liberal	27.2%	25.8%	28.0%	27.7%
Low Income (up to $60k)	40.6%	38.2%	42.3%	41.4%
High Income (over $60k)	59.4%	61.8%	57.7%	58.6%
No Bachelors Degree	63.1%	65.2%	63.0%	61.1%
Bachelors Degree	36.9%	34.8%	37.0%	38.9%
Metropolitan	85.1%	84.2%	85.0%	86.0%
Non-Metropolitan	14.9%	15.8%	15.0%	14.0%
Age (mean)	51.1	51.1	51.4	50.9
Young (up to 40)	30.7%	30.5%	30.8%	30.7%
Old (over 40)	69.3%	69.5%	69.2%	69.3%
Observations	1,802	600	600	602

Respondents were then informed that on the next few screens they would be asked to select a light bulb from two possible choices. They were also told that at the end of the survey they would be given a cash gift of $5.00 to purchase one of the light bulbs in the final choice set, which would be subsequently mailed to them. Though to reduce hypothetical bias respondents were told the options in the final choice set would depend on how they responded to the initial choices [30], the sixth choice set was randomly drawn from the available sets.

The difference between the price of the chosen light bulb and the amount of the cash gift was theirs to keep, so the final choice set entailed an actual purchase. S2 Table in S2 File shows that baseline model estimates are similar using only the stated preference data from the first five choice sets and the revealed preference data from the sixth choice set. Furthermore, we find no evidence of “straightlining,” where a respondent chooses the same response (usually the first) repeatedly to move quickly through a survey. The first and second options were chosen in 50.4% and 49.6% of the choices in the first five choice sets, respectively, and in 50.2% and 49.8% of choices in the sixth choice set, respectively.

**Fig 2:**
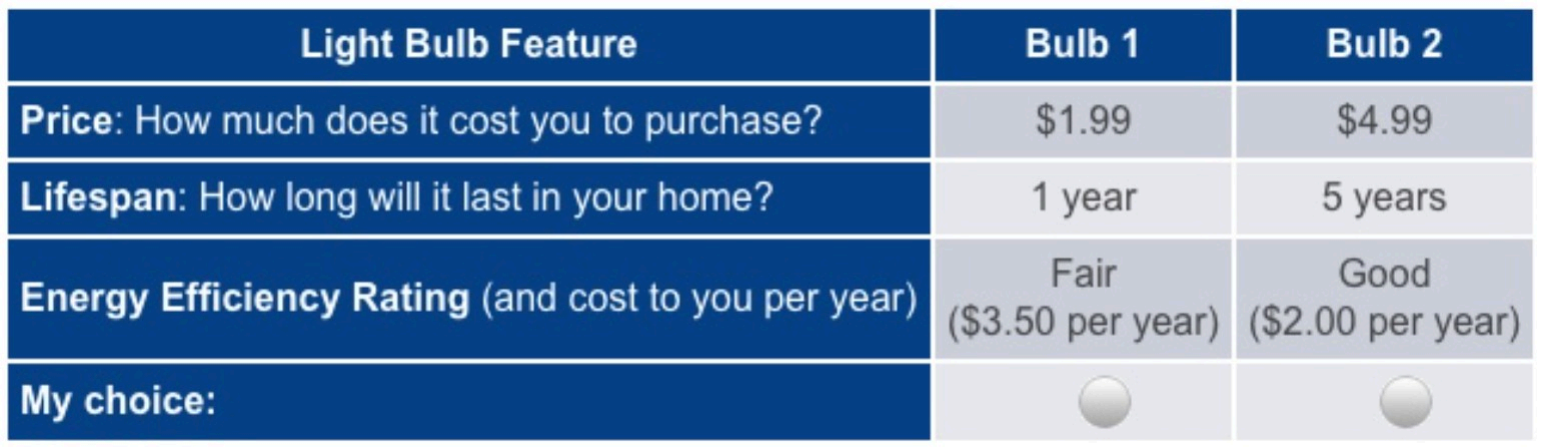
Light bulb choice set display.

Respondents were shown a total of six choice sets, each containing two light bulbs with varying values for price ($1.99, $2.99, $3.99, or $4.99), lifespan (1, 2, 5, or 10 years), and level of energy efficiency/intensity (1, 2, 3, or 4, corresponding to energy efficiency ratings of excellent, good, fair, or poor, respectively). While survey language refers to “energy efficiency,” with light bulb ratings of 1-excellent, 2-good, 3-fair, or 4-poor, we refer to this attribute as “energy intensity” in the analysis since a *higher* level is *more* energy intense. The attribute levels themselves were chosen to represent the typical ranges found in the marketplace, and the price levels in particular were chosen to span the full range of the $5.00 cash gift with which respondents were provided. In the case of lifespan and energy efficiency/intensity, the attribute levels were chosen to span the ranges indicated on the Energy.gov website at https://www.energy.gov/energysaver/save-electricity-and-fuel/lighting-choices-save-you-money/how-energy-efficient-light. Fig 2 shows an example of the choice set display as it was seen by respondents. Table 2 shows the summary statistics of the lightbulbs chosen by respondents according to treatment.

**Table 2:** Summary statistics of chosen light bulbs.

Treatment	Attribute	Obs.^†^	Mean	Std. Dev.	Min	Max
Frugality	Price	3,591	3.39	1.35	1.99	4.99
	Lifespan	3,591	3.63	2.99	1	10
	Energy Intensity	3,591	1.94	1.13	1	4
Environmental	Price	3,580	3.45	1.36	1.99	4.99
	Lifespan	3,580	3.54	2.95	1	10
	Energy Intensity	3,580	1.95	1.12	1	4
Energy Independence	Price	3,599	3.45	1.36	1.99	4.99
	Lifespan	3,599	3.61	3.00	1	10
	Energy Intensity	3,599	1.88	1.09	1	4

^†^Observations indicates the number of observed choice situations. Each of the 1,802 respondents was shown six choice sets, for a maximum total of 10,812 choice situations. Respondents were not forced to make a choice, resulting in a total number 10,770 observed choices.

To design the choice experiment, we used ChoiceMetrics’ Ngene software to generate a Bayesian D-efficient multinomial experimental design. This design would be efficient in the sense that it minimized the variance of the estimated coefficient for the utility function we pre-specified to underpin the multinomial logit model that we would fit to our data. To accomplish this, we employed a modified Federov algorithm in Ngene that permitted us to extract maximal information from our choice experiment by minimizing the variance-covariance estimator of the vector of coefficients for the specified utility function. We then selected the experimental design with the smallest D-error, defined as the determinant of the asymptotic variance-covariance matrix, that also contained no strictly dominated choices. We increased efficiency further by specifying Bayesian priors for the coefficient of each of the light bulb attributes in the specified utility function, because it has been shown that an experimental design’s efficiency is much improved even by defining the sign of those parameters [31]. We assumed negative a priori distributions for the coefficients on price and energy intensity (where higher is worse), because higher costs for either attribute would almost certainly decrease the likelihood that a bulb is chosen on average. Conversely, we assumed a positive a priori distribution for the coefficient on lifespan because longer light bulb longevity would almost certainly increase the likelihood that a bulb is chosen on average. This produced a design containing 60 choice sets from which each respondent’s 6 choice sets were drawn at random.

## 3 Model

The probability of consumer *i* selecting lightbulb *k* is the probability her utility from that lightbulb, *u*_*ik*_, is greater than her utility from choosing any other available alternative:

$$\begin{array}{c} \pi_{ik}=Prob(u_{ik}+\varepsilon_{ik}\geq u_{ij}+\varepsilon_{ij});\forall j\neq k. \end{array}$$ (1)

A linear utility function along with the assumption of type I extreme value errors (*ε*_*ik*_) allow us to model the probability of consumer *i* choosing lightbulb *k* as a mixed logit:

$$\begin{array}{c} \pi_{ik}=\int \frac{exp(\mu_{i}x_{k}^{\prime }\beta )}{\sum_{j=1}^{J}exp\left(\mu_{i}x_{j}^{\prime }\beta \right)}f\left(\beta |\theta \right)\partial \beta , \end{array}$$ (2)

where **x**_**k**_ is a vector of attributes of *k* and *β* is a vector of parameters, *μ*_*i*_ is a scale parameter commonly assumed to equal 1, and *f*(*β*|**θ**) is the density function of *β*.

Our baseline utility function is

$$\begin{array}{c} u_{ik}=\beta_{i1}price_{k}+\beta_{2}lifespan_{k}+\beta_{3}intensity_{k}+\beta_{4}intensity_{k}^{2}. \end{array}$$ (3)

The price coefficient, *β*_*i*1_ is random and assumed to be lognormally distributed, reflecting heterogeneity in price sensitivity with a long right tail. This is a common assumption for price variables in mixed logit models and tends to improve model fit relative to a normal distribution assumption that imposes equality of mean and median price sensitivity (e.g., [32]). The lifespan and energy intensity parameters are assumed to be fixed. Attempts to specify them as random parameters lead to numeric instability in the model estimation such that it does not converge to a well-defined maximum value.

In a multinomial logit (with all fixed parameters), willingness to pay (WTP) for a marginal change in an attribute can be calculated by dividing the attribute’s coefficient by the negative of the price coefficient. In a mixed logit with random parameters, WTP can be estimated via simulation by taking random draws from the parameter distributions in the calculation of this ratio.

In some specifications energy intensity is interacted with consumer characteristics (e.g., income) and/or treatment (e.g., environmental) to allow for heterogeneity in preferences across these dimensions. Of the 1,802 person sample, 37.7% are conservative, 35.1% liberal; just under sixty percent have incomes over $60,000, 36.9% have a Bachelors degree or higher, 85% live in metropolitan areas, and the mean age is 51.

## 4 Results

### 4.1 Heterogeneity of energy efficiency preferences and externality-focused messaging

Choice model estimation results (estimated in WTP space) are shown in Table 3. The non-price coefficients represent marginal WTP calculated via simulation as part of the Stata *mixlogitwtp* package to estimate mixed logit models. Specifying WTP distributions at the estimation stage leads to better data fit than taking coefficient ratios post estimation [33]. Given power constraints, the specifications in Table 3 are estimated using the full sample- including respondents who were treated with each of the three treatments. The coefficients should therefore be interpreted as average preferences across treatment groups. Table 4 includes interactions between treatment and energy intensity preferences. Interactions between treatment and the other attributes (price and lifespan) are not statistically significantly different across treatment.

**Table 3:** Willingness to pay for energy intensity.

	(1)	(2)	(3)	(4)	(5)	(6)	(7)	(8)
	Full Sample		Political Identity		Income	Education	Metro	Age
-Price (mean)	-1.255***	-1.161***	-1.308***	-1.308***	-1.482***	-1.367***	-1.205***	-1.232***
	(0.061)	(0.077)	(0.002)	(0.002)	(0.113)	(0.086)	(0.071)	(0.076)
-Price (stdev)	1.902***	2.295***	1.857***	1.903***	1.049***	1.442***	1.992***	1.870***
	(0.000)	(0.000)	(0.006)	(0.001)	(0.170)	(0.022)	(0.000)	(0.173)
Lifespan	0.577***	0.582***	0.581***	0.580***	0.645***	0.608***	0.588***	0.554***
	(0.019)	(0.016)	(0.000)	(0.016)	(0.050)	(0.027)	(0.012)	(0.036)
Intensity	-3.049***	-1.912***						
	(0.019)	(0.320)						
Intensity^2^		-0.213***	-0.241***	-0.243***	-0.224***	-0.285***	-0.219***	-0.178***
		(0.058)	(0.022)	(0.048)	(0.067)	(0.060)	(0.048)	(0.057)
Intensity*Conservative			-1.552***^,*a*^					
			(0.162)					
Intensity*Liberal			-2.888***^,*b*^	-2.884***^,*d*^				
			(0.164)	(0.249)				
Intensity*Non			-1.644***^,*c*^					
			(0.000)					
Intensity*Conservative or Non				-1.585***^,*e*^				
				(0.280)				
Intensity*Low					-1.844***^,*f*^		
						(0.357)		
Intensity*High					-2.472***^,*g*^			
					(0.415)			
Intensity*No Bachelors						-1.459***^,*h*^		
						(0.354)		
Intensity*Bachelors						-2.297***^,*i*^		
						(0.470)		
Intensity*Non Metro							-1.748***^,*j*^	
							(0.305)	
Intensity*Metro							-1.892***^,*k*^	
							(0.284)	
Intensity*Old								-2.113***^,*l*^
								(0.379)
Intensity*Young								-1.874***^,*m*^
								(0.347)
Observations^†^	10,770	10,770	10,770	10,770	10,770	10,770	10,770	10,770
AIC	11,436	11,408	11,357	11,356	11,427	11,395	11,411	11,411
BIC	11,468	11,448	11,413	11,404	11,475	11,443	11,459	11,459
T-test between			*a*, *b*	*d*, *e*	*f*, *g*	*h*, *i*	*j*, *k*	*l*, *m*
p-value			0.0000	0.0000	0.0117	0.0049	0.5915	0.1237
T-test between			*a*, *c*					
p-value			0.5688					

Standard errors in parentheses are clustered at the respondent level.

*** p<0.01,

** p<0.05,

* p<0.1.

^†^Observations indicates the number of observed choice situations. Each of the 1,802 respondents was shown six choice sets, for a maximum total of 10,812 choice situations. Respondents were not forced to make a choice, resulting in a total number 10,770 observed choices. “Liberal” consumers identify as “very,” “somewhat,” or “closer to” liberal. “Conservative” consumers identify as “very,” “somewhat,” or “closer to” conservative. “Non” political consumers identified neither as liberal nor conservative or declined to answer the question on political identity. “Low” and “High” income consumers refer to those making up to or more than $60,000 a year, respectively. “Old” and “Young” consumers refer to those who are up to or over 40 years of age, respectively. Note that only lightbulb attributes that vary across alternatives can be included in the mixed logit estimation. Consumer characteristics on their own are dropped from estimation due to collinearity and can only be included as interactions with lightbulb attributes. Superscripts *a*-*m* serve as coefficient labels to indicate which coefficients are compared in the t-tests in the last four rows of the table.

**Table 4:** Impacts of Externality-Focused Messaging on WTP.

	(1)	(2)	(3)	(4)	(5)	(6)
	Full Sample	Political Identity	Income	Education	Metro	Age
-Price (mean)	-1.139***	-1.285***	-1.171***	-1.287***	-1.217***	-1.101***
	(0.096)	(0.481)	(0.082)	(0.101)	(0.081)	(0.085)
-Price (stdev)	2.217***	2.013***	2.159***	1.818***	1.980***	2.353***
	(0.080)	(0.031)	(0.001)	(0.366)	(0.224)	(0.000)
Lifespan	0.586***	0.581***	0.574***	0.573***	0.560***	0.573***
	(0.012)	(0.037)	(0.025)	(0.026)	(0.035)	(0.035)
Intensity^2^	-0.205***	-0.291***	-0.191**	-0.238***	-0.181**	-0.213***
	(0.032)	(0.067)	(0.083)	(0.045)	(0.079)	(0.035)
Intensity*Frugality^a^	-1.556***	-1.158***	-1.417***	-1.357***	-1.821***	-1.480***
	(0.171)	(0.389)	(0.396)	(0.265)	(0.435)	(0.215)
Intensity*Environmental^b^	-1.926***	-1.325***	-1.985***	-1.712***	-2.117***	-1.947***
	(0.219)	(0.368)	(0.436)	(0.331)	(0.422)	(0.248)
Intensity*Energy Independence^c^	-2.300***	-1.447**	-2.302***	-2.018***	-2.394***	-2.427***
	(0.378)	(0.695)	(0.456)	(0.316)	(0.522)	(0.201)
Intensity*Frugality*Liberal		-0.305				
		(0.292)				
Intensity*Environmental*Liberal		-1.296***				
		(0.244)				
Intensity*Energy Indep*Liberal		-3.247				
		(14.520)				
Intensity*Frugality*High			-0.552***			
			(0.133)			
Intensity*Environmental*High			-0.116			
			(0.301)			
Intensity*Energy Indep*High			-0.240			
			(0.480)			
Intensity*Frugality*Bachelors				-0.490		
				(0.353)		
Intensity*Environmental*Bachelors				-0.291		
				(0.351)		
Intensity*Energy Indep*Bachelors				-1.213***		
				(0.417)		
Intensity*Frugality*Metro					-0.00994	
					(0.508)	
Intensity*Environmental*Metro					0.168	
					(0.204)	
Intensity*Energy Indep*Metro					-0.0318	
					(0.355)	
Intensity*Frugality*Young						-0.282**
						(0.118)
Intensity*Environmental*Young						0.234
						(0.194)
Intensity*Energy Indep*Young						0.631
						(0.474)
Observations^†^	10,770	10,770	10,770	10,770	10,770	10,770
AIC	11,391	11,321	11,383	11,379	11,400	11,381
BIC	11,447	11,401	11,463	11,459	11,480	11,461
AIC	11,391	11,321	11,383	11,379	11,400	11,381
BIC	11,447	11,401	11,463	11,459	11,480	11,461
P-value of t-test						
between a,b	0.0026	0.2685	0.0014	0.0488	0.5279	0.0000
between a,c	0.0387	0.4066	0.0000	0.0010	0.2738	0.0085
between b,c	0.3150	0.7508	0.0867	0.2266	0.3323	0.1995

Standard errors in parentheses are clustered at the respondent level.

*** p<0.01,

** p<0.05,

* p<0.1.

^†^Observations indicates the number of observed choice situations. Each of the 1,802 respondents was shown six choice sets, for a maximum total of 10,812 choice situations. Respondents were not forced to make a choice, resulting in a total number 10,770 observed choices. “Liberal” consumers identify as “very,” “somewhat,” or “closer to” liberal. “Conservative” consumers identify as “very,” “somewhat,” or “closer to” conservative. “Non” political consumers identified neither as liberal nor conservative or declined to answer the question on political identity. “Low” and “High” income consumers refer to those making up to or more than $60,000 a year, respectively. “Old” and “Young” consumers refer to those who are up to or over 40 years of age, respectively. Note that only lightbulb attributes that vary across alternatives can be included in the mixed logit estimation. Consumer characteristics on their own are dropped from estimation due to collinearity and can only be included as interactions with lightbulb attributes.

The price coefficient is assumed to be random and lognormally distributed (and hence the negative must enter the utility function), reflecting heterogeneity in price sensitivity with a long right tail. Columns 1 and 2 show the baseline specification, with Column 2 including a quadratic energy intensity term that improves model fit (as evidenced by the lower Akaike and Bayesian information criteria) and suggests concavity in the value function for energy intensity. Across the different specifications, consumers are willing to pay just under $0.6 for an additional year of lifespan and -$2–3 (with considerable heterogeneity) per unit increase in energy intensity. Columns 3 through 8 include interactions between energy intensity and consumer political identity, education, metropolitan status, and age. These characteristics are chosen because they are fairly standard demographic variables that are more easily observable than underlying attitudinal variables. The intention here is to show that energy efficiency preferences do vary across standard consumer demographics. Liberal consumers have a lower willingness to pay for energy intensity (higher WTP for energy efficiency), as do higher income consumers (making over $60,000 per year), more educated consumers (with a Bachelors degree), and older consumers (over 40 years of age).

Table 4 includes triple interactions between energy intensity, consumer characteristics, and treatment (energy independence and environmental messaging) and shows evidence of differential responsiveness to the three treatments. Interactions between treatment and lifespan are not statistically significant. When interactions between treatment and price are included, the model becomes numerically unstable and the log pseudolikelihood does not converge to a well-defined maximum value. The interactions between energy intensity and the three treatments in Column 1 show that overall, consumers receiving the energy independence message have the lowest WTP for energy intensity (-$2.3), followed by those receiving the environmental message (-$1.9). The coefficients on the energy intensity interactions with the environmental and energy independence treatments are each statistically significantly different from the interaction between energy intensity and the baseline frugality treatment, with two-sided t-test p-values of 0.0026 and 0.0387, respectively, although the difference between the energy intensity interaction with the environmental treatment is not significantly different from that of the energy independence treatment.

Just as Table 3 demonstrates that energy efficiency preferences vary across standard consumer demographics, Columns 2 through 6 of Table 4 demonstrate that responsiveness to the alternative moral suasion treatments varies across demographics. For example, the environmental message induces lower WTP for energy intensity by liberals, while the energy independence message induces lower WTP for energy intensity by consumers with a bachelors degree. The results in Table 4 suggest that not only can externality-focused messages induce consumers to make more energy efficient purchases, but some messages are more impactful on certain consumers than others. This in turn suggests that messages could be targeted by consumer demographics. As in Table 3, the consumer characteristics used in Table 4 were chosen because they are fairly standard and relatively easily observable. Note that we do not necessarily identify the most salient source of heterogeneous responsiveness or address fundamental causes of the heterogeneous responsiveness. The purpose is to demonstrate that messages could be targeted along standard and observable sources of heterogeneity. Note that the machine learning algorithm explored in Section 4.3 does identify the most important sources of heterogeneity driving responsiveness to the alternative externality-focused messaging treatments.

Results are consistent with the related literature. [20] also finds that environmental priming has a significant and positive impact on WTP for LEDs, at least in some cases. However, political affiliation is not included in the study. That liberals are more likely and conservatives less likely to purchase a more energy efficient good when environmental benefits are highlighted is consistent with the literature ([15, 34–37]).

### 4.2 Policy implications

In this section we investigate the effectiveness of alternative subsidy programs and messaging campaigns. The simulated policies are summarized in Table 5, which shows energy intensity of light bulbs chosen by the representative sample for each policy. Counterfactual 0 (Cf0) in the first row is a baseline policy, in which every consumer receives the frugality message. Under this policy, mean energy intensity of light bulbs selected by the sample is 1.973. Recall the lowest energy intensity is 1 (excellent energy efficiency) and the highest is 4 (poor energy efficiency). Thus, the mean energy intensity of 1.973 is fairly good. The next four columns show the percent of the sample choosing light bulbs of each possible energy efficiency rating.

**Table 5:** Counterfactual policy simulations.

		Mean Energy Intensity	Bootstrapped		Percent Choosing			Relative Energy Consumption^†^
			Std. Error	Excellent (1)	Good (2)	Fair (3)	Poor (4)	
Cf0	Frugality Message	1.973	0.016	49.6%	21.2%	12.2%	17.4%	100.0%
**Uniform Policies**								
Cf1	$1 Subsidy for Excellent	1.936	0.011	51.6%	20.5%	11.6%	16.7%	98.2%
Cf2	Environmental Message	1.929	0.005	50.9%	21.5%	12.1%	15.9%	97.5%
Cf3	Energy Indep Message	1.892	0.009	52.0%	21.8%	12.1%	14.5%	95.4%
**Targeted Policies**								
Cf4	$1 Subsidy for Excellent to Low Income^††^	1.963	0.013	50.3%	20.8%	12.0%	17.3%	99.6%
Cf5	Environmental to Liberals	1.931	0.015	50.8%	21.4%	12.5%	15.7%	97.6%
Cf6	Energy Indep to Bachelors	1.920	0.008	51.2%	21.4%	12.3%	15.5%	97.0%

Six counterfactual policies (Cf1-Cf6) are simulated and compared in terms of mean energy efficiency of chosen light bulbs and percentage of consumers choosing light bulbs with excellent through poor energy efficiency ratings. Subsidy policies Cf0—Cf3 are generated using estimates from Column 1 of Table 4. Policies Cf4, Cf5, and Cf6 are generated using estimates from Columns 3, 2, and 4 of Table 4, respectively. The average lightbulb price in the choice experiment was $3.43, and the average price of a light bulb with excellent energy efficiency was $3.58.

^†^Poorly rated light bulbs are assumed to have an energy factor of 1. Light bulbs with fair, good, and excellent ratings are assumed to have energy factors of 0.75, 0.35, and 0.25. These factors are based on the efficiency levels shown on https://www.energy.gov/energysaver/save-electricity-and-fuel/lighting-choices-save-you-money/how-energy-efficient-light, which were used to determine efficiency attribute levels in the experimental design.

^††^Low income consumers are defined as having annual incomes up to $60,000. Two-sided t-tests for differences in means show mean efficiency in Cf0 is statistically significantly different from Cf1, with a p-value of 0.0435, and Cf1 is significantly different from Cf3, with a p-value of 0.0014. Cf1 is not significantly different from Cf2, though Cf2 is significantly different from Cf3, with a p-value of 0.0002.

Policy Cf1 provides a $1 subsidy for lightbulbs with an “excellent” energy efficiency rating (i.e., lowers the price of these bulbs by $1 and leaves prices of other-rated light bulbs unchanged). This increases the share of the sample who chooses the most energy efficient bulbs to 51.6% from 49.6%, lowering mean intensity to 1.936 from 1.973. Note that since policy makers could not distinguish between additional and non-additional consumers, this subsidy would be given not only to the additional 3.9% of consumers induced by the subsidy to purchase the excellent rated bulb, but also to the 49.6% of infra-marginal consumers who would have chosen the bulb even without the subsidy. Hence, the subsidy is likely to require large amounts of public revenue per decrease in energy consumption. Indeed, within our representative sample, the total cost of the subsidy would be $926 and result in only 35 additional excellent-rated bulb purchases, resulting in a cost of $26.4 per additional excellent-rated bulb purchase. This is equivalent to a social cost of carbon (SCC) of approximately $183. The SCC calculation is based on energy factors taken from energy.gov, assume the poor rated bulb is a standard 60W incandescent, assume a lightbulb life of 25,000 hours, and assume carbon intensity of 0.92 pounds of CO2 per kWh, the 2019 national average according to https://www.eia.gov/tools/faqs/faq.php?id=74&t=11. The calculation also takes into account the rating of the bulb each consumer would otherwise have purchased. A two-sided t-tests reveals mean efficiency in Cf0 is statistically significantly different from Cf1, with a p-value of 0.0435.

Policies Cf2 and Cf3 in Table 5 display results of uniform moral suasion messaging policies. Rather than provide subsidies, the policy maker messages each consumer with the environmental message (Cf2) or the energy independence message (Cf3), promoting energy efficiency by highlighting negative externalities associated with energy consumption. When everyone is treated with the environmental message (Cf2), the share of consumers purchasing excellent-rated bulbs increases somewhat (to 50.9% from 49.6%). The percent of consumers choosing “good” efficiency bulbs increases slightly, and the share of those choosing bulbs rated as “fair” or “poor” decreases. Mean intensity declines to 1.929 from 1.973, a smaller improvement than that associated with the Cf1 subsidy. T-tests for differences in means show mean intensity in Cf1 is significantly different from Cf3, with a p-value of 0.0014, and mean intensity in Cf2 is significantly different from Cf3, with a p-value of 0.0002. However, mean intensity in Cf1 is not significantly different from Cf2.

When all consumers receive the energy independence message in Cf3 of Table 5, mean energy intensity improves significantly, to 1.892 from 1.973. The share of consumers choosing the most energy efficient bulbs increases to 52.0% from 49.6%. The share choosing “good” also increases, while the share choosing the worst-rated bulbs (“poor”) decreases substantially, to 14.5% from 17.4%. The subsidy (Cf1) mostly shifts consumers who would otherwise purchase “good” rated lightbulbs to purchasing “excellent” rated bulbs because they “close the gap” in WTP for efficiency first for these consumers. Subsidies are less likely to close the gap in WTP for consumers choosing “poor” rated bulbs. The externality-focused messaging, especially Cf3, also shifts more consumers into choosing “good” rated bulbs rather than “fair” or “poor” rated bulbs. While the improvements in energy intensity levels of the various counterfactuals in Table 5 might seem incremental, these would lead to non-trivial energy savings. The last column of Table 5 shows the energy consumption of Cf1-Cf6 relative to Cf0 based on energy factors from energy.gov. Cf3 reduces energy consumption by 4.6% relative to Cf0.

Policies Cf4-Cf6 are targeted towards certain demographics. Cf4 provides the $1 subsidy for “excellent” rated bulbs only to lower income consumers (who make up to $60,000 per year). Various existing energy policies assign larger incentives to lower income households on grounds of equity and cost-effectiveness, since these households are often more price sensitive. Examples of such low-income focused policies include the Clean Cars for All, the Low Income Weatherization Program, and the Low Income Household Energy Assistance Program, among others. Cf4 lowers mean intensity and reduces electricity consumption only slightly relative to Cf0 and substantially less than Cf1 (the uniform subsidy). This is due to two reasons. First, further modeling efforts interacting income with price suggest no significant difference in price elasticity of demand between high and low income consumers when it comes to light bulb purchases. Second, although there are fewer infra-marginal consumers in Cf4- the subsidy is only given to the low income consumers who make up 40% of excellent-rated bulb buyers- there are even fewer marginal buyers under this policy because low income consumers have a significantly lower WTP for efficiency. The $1 subsidy is not as effective at closing the WTP gap for these individuals. The total subsidy cost for the representative sample would be $373 but would only result in 12 additional excellent-rated bulb purchases, at a cost of $31.5 per additional lightbulb. This is equivalent to a social cost of carbon (SCC) of roughly $278.

Policy Cf5 messages only liberals with the environmental message and Cf6 messages only more educated consumers (with Bachelors degrees) with the energy independence message (and everyone else with the frugality message). Cf5 and Cf6 both lower energy intensity more than the $1 subsidies, with mean energy intensity of 1.931 and 1.920, respectively. Notably, targeting the environmental message only to the liberal subset of consumers (Cf5) improves intensity almost as much as treating the entire sample with the environmental message (Cf2), with a mean intensity of 1.931 versus 1.929. Assigning the energy independence message only to individuals with a Bachelors degree (Cf6) also accounts for a majority of the energy efficiency gains in Cf3 relative to Cf0. Together, Cf5 and Cf6 suggest that targeting messages towards particular consumer segments can lead to large energy efficiency improvements. In Section 4.3 we explore how to increase efficiency further by targeting different consumer types with different messages.

Overall, this policy comparison suggests that externality-focused messaging could be a more cost effective policy to promote energy efficiency relative to subsidies. It is also worth reiterating that subsidies tend to operate on the upper end of the energy efficiency distribution (converting “good” bulb purchases to “excellent”) while suasion messaging also operates at the lower end of the energy efficiency distribution, inducing more “poor” bulb purchasers to upgrade.

### 4.3 Micro-targeting

#### 4.3.1 Optimal micro-targeting

Using machine learning, we explore the possibility of “micro-targeting” the moral suasion messaging, i.e., targeting each consumer with the message that will induce the most energy efficient purchase by the consumer. We use supervised machine learning to predict energy intensity decisions for each consumer under each of the three possible treatments (frugality, environmental, and energy independence message) based on a large set of consumer characteristics. Having obtained predictions for all consumers under each of the three treatments, we micro-target by assigning each consumer the “optimal” message- that which leads to the most energy efficient (lowest energy intensive) purchase.

Specifically, we use the elastic net algorithm, a regularized machine learning model. The elastic net nests the least absolute shrinkage and selection operator (lasso) and ridge regression estimations, solving an ordinary least squares objective function penalizing variables with large coefficients and setting irrelevant coefficients to zero [38]. This avoids overfitting and can lead to substantial decreases in out of sample mean squared error relative to standard regression methods.

First, for each treatment, we train a model on the subsample that actually received that treatment. Then, we apply the model to the other consumers. For example, we train a model to estimate energy intensity decisions on consumers who received the frugality message. Then we use the model on consumers who received the environmental or energy independence treatment to predict their energy intensity decision had they instead been treated with the frugality message. We repeat this for the environmental and energy independence messages. Each consumer is then assigned the “optimal” treatment that results in the most energy efficient light bulb purchase.

In the machine learning models we include all 63 consumer characteristics gathered in the survey, including demographic and socioeconomic characteristics, as well as attitudinal variables and indicators for recent political involvement and support of various organizations (e.g., the National Rifle Association and the Sierra Club). The covariates that are most influential on consumer energy intensity choices in the elastic net model include basic demographics, such as marital status, employment status, income, and education, as well as political party and various political beliefs/activities. A complete list of covariates and the twenty most influential are shown in S3, S4 Tables in S1 File.

In the optimal micro-targeting policy, each consumer is assigned the treatment that results in the most energy efficient (lowest energy intensive) light bulb purchase. Panel A of Table 6 shows that under optimal micro-targeting, 23% of consumers receive the environmental message, 36% the energy independence, and 42% the frugality. Under optimal micro-targeting, mean energy intensity is 1.79, substantially lower than that of any of the homogeneous messaging policies (i.e., if all consumers were given either the frugality, environmental, or energy independence message), which varies from 1.95 (environmental) to 1.88 (energy independence). The predicted mean energy efficiencies for uniform messaging differs from those in Table 5 given both the alternative estimation methods and covariates. For each of the designs (i.e., frugality message, environmental message, energy independence message, and optimal micro-targeting), a difference in means test reveals the mean energy intensity is significantly different between each pair of designs at the 5% significance level except between the frugality and energy independence messages.

**Table 6:** Micro-targeting externality-focused messaging.

**Panel A: Comparison of targeting designs**						
	Mean Energy Intensity	Bootstrapped	Percent Receiving each Treatment			Relative Energy Consumption^†^
		Std. Error	Frugality	Environmental	Energy Independence	
All Covariates						
Frugality Message	1.91	0.004	100%			100.0%
Environmental Message	1.95	0.007		100%		101.8%
Energy Independence Message	1.88	0.033			100%	98.5%
**Optimal Micro-targeting**	1.79	0.004	42%	23%	36%	94.3%
Top 20 Covariates						
Frugality Message	1.93	0.009	100%			100.0%
Environmental Message	1.95	0.020		100%		101.1%
Energy Independence Message	1.88	0.031			100%	97.5%
**Micro-targeting**	1.84	0.005	34%	9%	57%	95.9%
Top 10 Covariates						
Frugality Message	1.94	0.027	100%			100.0%
Environmental Message	1.95	0.023		100%		100.4%
Energy Independence Message	1.87	0.013			100%	96.9%
**Micro-targeting**	1.86	0.003	25%	2%	73%	96.1%
**Panel B: Micro-targeting out of sample performance** ^††^						
Micro-targeting Method	Covariates	Predicted Mean Intensity (Out of Sample)		Actual Mean Intensity		Error
Elastic Net	All	1.916		1.915		0.05%
Regression (OLS)	All	1.907		1.915		0.46%
Elastic Net	Top 20	1.917		1.915		0.08%
Elastic Net	Top 10	1.917		1.915		0.06%

The full set of covariates as well as the top 20 with the largest impact out of the full set of covariates in the elastic net model on consumer energy intensity choices are shown in the supporting information.

^†^Poorly rated light bulbs are assumed to have an energy factor of 1. Light bulbs with fair, good, and excellent ratings are assumed to have energy factors of 0.75, 0.35, and 0.25. These factors are based on the efficiency levels shown on https://www.energy.gov/energysaver/save-electricity-and-fuel/lighting-choices-save-you-money/how-energy-efficient-light, which were used to determine efficiency attribute levels in the experimental design. An exponential function is fit to these discrete energy factors to allow for interpolation. The equation of the fitted function is *energy use factor* = 0.1479*e*^0.4921*efficiency rating^, and the R-squared of the fit is 0.97.

^††^ For each method, two-thirds of the respondents in the sample are randomly selected to estimate/train the model. Estimation results are used to predict intensity level of light bulbs chosen by the remaining one-third of the respondents in the sample. The “Actual Mean Intensity” is the actual mean energy intensity level chosen by the one-third subsample. The “Predicted Mean Intensity” is the mean energy intensity level predicted for the one-third subsample by each method. “Error” is the percentage difference between the “Predicted Mean Intensity” and the “Actual Mean Intensity” For each of the designs using all covariates (i.e., frugality message, environmental message, energy independence message, and optimal micro-targeting), a difference in means test reveals the mean energy intensity is significantly different between each pair of designs at the 5% significance level except between the frugality and energy independence messages. The difference in means between the optimal micro-targeting design using all covariates is significantly different at the 1% significance level from both the micro-targeting design using the top 20 and the top 10 covariates.

#### 4.3.2 Out of sample model performance

To assess the performance of the elastic net model, we perform a simple exercise to compare its out of sample performance to that of standard OLS regression. Two-thirds of the respondents in the sample are randomly selected on whom to train the elastic net model and to estimate the OLS regression. Estimation results are used to predict intensity level of light bulbs chosen by the remaining one-third of the respondents in the sample. The same full set of covariates is utilized in each model. Panel B of Table 6 displays the results. The “Actual Mean Intensity” is the actual mean energy intensity level chosen by the one-third subsample. The “Predicted Mean Intensity” is the mean energy intensity level predicted for the one-third subsample by each method. “Error” is the percentage difference between the “Predicted Mean Intensity” and the “Actual Mean Intensity.” The first and second rows show that this out of sample error in estimated mean intensity is 0.05% for the elastic net model and 0.46% for the OLS regression, implying that the elastic net model out of sample performance is an order of magnitude better than a standard regression.

#### 4.3.3 Limited covariates

Our results show that optimal micro-targeting can substantially improve consumer energy efficiency decisions relative to uniform suasion messaging. However, one barrier to implementing a real world micro-targeting campaign is the amount of consumer information required. To investigate performance of micro-targeting with a more limited set of information, we re-run the elastic net methodology using smaller sets of covariates- the top twenty and the top ten most influential covariates from the full model. The bottom two sub-sections of Table 6, Panel A show policy performance of uniform and micro-targeting based on elastic net models using these two more limited sets of covariates. Micro-targeting using the top twenty covariates results in mean energy intensity of 1.84, a substantial improvement over that of the best-performing uniform message, energy independence (1.88). Micro-targeting using the top ten covariates results in a smaller improvement over uniform energy independence messaging (1.86 versus 1.87). The reduction in energy consumption depends on the distribution of poor, fair, good, and excellent light bulbs, which is not predicted by the elastic net method (rather, a level of energy intensity on a continuous spectrum is estimated). However, as shown in Table 5, a 0.01 improvement in mean intensity can be associated with more than a 0.5% decrease in energy consumption, and a 0.04 improvement in mean intensity with around a 2% decrease in energy consumption. As the number of covariates decreases from all to twenty to ten, the percent of respondents receiving the environmental treatment decreases and the percent of those receiving the energy independence increases. This may suggest it is more difficult to identify consumers who respond best to the environmental treatment.

Although micro-targeting policy performance appears to be superior to uniform messaging, we must also verify that the predictive power of the covariate-limited models remains acceptable. We repeat the out of sample test on the elastic net models using the top twenty and top ten covariates. The third and forth rows of Table 6, Panel B shows out of sample error in predicted mean intensity of 0.08% and 0.06%, respectively- slightly higher than using the full set of covariates, but the same order of magnitude. Therefore, we conclude that to a certain extent, utilizing machine learning for micro-targeting can still be effective using a limited set of consumer information without sacrificing predictive power.

It would likely be difficult for a policy maker to gather all of the top twenty variables at the individual level in order to implement micro-targeting. However, many of the variables, including housing structure type and household income, could be gathered at the census block level. Political affiliations can also be inferred from recent election results. Some of the organization support variables (e.g., supports Greenpeace) could potentially be shared at an aggregate level by the organizations themselves. Thus, in practice, it would likely be feasible to gather enough (e.g., 10) variables at an aggregate level allowing for micro-targeting at the aggregate (e.g., census block) level.

Ideally, for real world implementation of a micro-targeting program, policy makers would first implement a small scale pilot study, during which many variables are gathered and tested. Policy makers could then determine which subset of variables are both impactful and possible to collect for use in micro-targeting at a broad scale.

## 5 Discussion and conclusion

These findings deepen our understanding of the comparative performance of policy interventions that are designed to internalize consumption externalities. First, we provide the first comparative analysis for a popular type of second-best subsidy for a best-in-class energy efficient durable good. Nearly all of the prior research examined the effects of alternative policy interventions for consumers who continuously purchase a commodity on a daily basis, such as energy [1, 2]. The episodic nature of durable goods purchases may mean that consumers will become less habituated to suasion messages, making them more effective, as compared to continuously-purchased commodities such as electricity, water, natural gas or transportation fuels.

Second, we find that uniformly-targeted suasion messages appear to shift the entire distribution of consumers’ preferences for energy efficiency. In contrast, the second-best subsidy, which could be applied only to those light bulbs rated “excellent” for energy efficiency, increases the willingness to pay primarily of consumers who previously preferred light bulbs with a “good” rating but did not significantly influence the choices of those who had selected “fair” or “poor” rated light bulbs. The broader scope of influence associated with suasion messages appears to explain much of their superior performance relative to the second-best subsidy considered here.

Third, our analysis reveals that suasion messages differ greatly in their productivity regardless of whether they are uniformly-administered or micro-targeted. Therefore, deepening our understanding of message-based psychological mechanisms that influence consumers’ willingness to pay appears increasingly important for economist engaged in the design of policy interventions. Given the simplicity of messages used by economists to date, the opportunities for innovation appear quite large.

Fourth, our central contribution is to illuminate the potential efficiency gains that micro-targeting suasion messages yields. In the presence of heterogeneous messages and consumers, micro-targeting can significantly enhance the internalization of externalities. It may also do so more cost effectively than would occur if researchers ignore consumer heterogeneity. Recall that we find that for even the uniformly-assigned suasion messages, much of their overall effectiveness is associated with the response of only a subset of consumers. Therefore, our ability to selectively target uniform messages to only a subset of customers could enhance the cost effectiveness of this policy intervention without significantly diminishing their ability to spur greater willingness to pay for energy efficiency.

Finally, although micro-targeting is becoming more feasible as consumers increasingly search and make purchases in digital retail environments, our study nonetheless explores way to minimize the ex ante informational costs associated with consumer segmentation and micro-target models. Policy makers do need to first collect somewhat detailed information from a subset of consumers, but we show how this intervention could then be scaled up for broader deployment using less consumer information that is more easily collectable or observable.

## Supporting information

S1 File Additional tables of summary statistics and results. (PDF)

S2 File (DTA)
