# Supplementary material for: Micro-targeting consumers to reduce consumptive externalities
Source: PLoS One. 2023 May 4;18(5):e0284338. doi: 10.1371/journal.pone.0284338 (PMC10159161; doi:10.1371/journal.pone.0284338)
Supplement: S1 File — (PDF) [file pone.0284338.s001.pdf]

Supporting Information

Table S1. Demographics in Representative Sample versus U.S. Population

Demographic category	Representative sample	U.S. population
**Age**		
18-35	23.6	32.3
36-65	54.0	48.9
Over 65	22.4	18.9
**Sex**		
Female	51.8	50.8
Male	48.2	49.2
**Race**		
White	65.0	61.3
African American	11.7	13.3
Hispanic	15.6	17.8
Other	7.7	7.1
**Educational attainment**		
No degree	10.1	11.7
High school degree	57.6	48.1
Bachelor or higher	32.3	40.2
**Region**		
Northeast	17.9	17.4
South	37.6	37.9
Midwest	21.2	21.0
West	23.4	23.7

Table S1 compares the demographics of our representative sample of 1,802 voting-age U.S. citizens to those of the U.S. population as a whole. All population demographics reported are percentage estimates from the U.S. Census Bureau for the total U.S. population with the exception of the age and educational attainment estimates, which represent percentages of the U.S. population over the age of 18 to ensure that the sample and population figures for these demographic categories are directly comparable.[1]

Table S2. Comparison of Stated Preference to Revealed Preference Choice Sets

	(1) Stated Preference	(2) Revealed Preference
-Price (mean)	-1.189***	-0.868***
	(0.000)	(0.150)
-Price (stdev)	2.337***	1.676***
	(0.000)	(0.424)
Lifespan	0.590***	0.710***
	(0.009)	(0.107)
Intensity	-2.103***	-1.067***
	(0.363)	(0.324)
Intensity^2	-0.166**	-0.373***
	(0.069)	(0.053)
Observations	17,946	3,594
AIC	9,623	1,780
BIC	9,662	1,811

Table S2 compares the baseline choice model estimates using the stated preference (SP) choice sets to the estimates using the revealed preference (RP) choice sets (i.e., the final choice set in the choice experiment in which the respondent purchased his/her preferred bulb). Standard errors in parentheses are clustered at the respondent level. *** p<0.01, ** p<0.05, * p<0.1.

Table S3. Covariates used in Machine Learning

Partner's Political Identity
Could use common 60W light bulb in home
Registered to Vote
Votes in Local Elections
Importance of protecting environment in purchase decision
Importance of acting frugally in purchase decision
Importance of acting patriotically in purchase decision
Supportive of Trump Administration
Political Party
Political Ideology
Voted for whom in 2016 Presidential election
Interest in politics/public affairs
Attended political protest or rally in past year
Contacted a government official in past year
Volunteered/worked for Presidential campaign in past year
Volunteered/worked for political candidate/cause in past year
Served on a committee for civic, nonprofit, community organization in past year
Written to newspaper/magazine or called radio/TV show in past year
Shared opinion about town issue at public meeting in past year
Commented on politics on internet in past year
Held publicly elected office in past year
Signed petition in past year
Identifies with/actively supports Tea Party
Identifies with/actively supports Environmental Rights
Identifies with/actively supports Women's Rights
Identifies with/actively supports Racial Equality
Identifies with/actively supports Right to Life
Identifies with/actively supports Peace
Identifies with/actively supports LGBTQ Rights
Identifies with/actively supports Indivisible
Identifies with/actively supports Black Lives Matter
Identifies with/actively supports NRA
Identifies with/actively supports Heritage Foundation
Identifies with/actively supports Planned Parenthood
Identifies with/actively supports National Right to Life Committee
Identifies with/actively supports Greenpeace
Identifies with/actively supports Sierra Club
Identifies with/actively supports Amnesty International
Identifies with/actively supports National Education Association Foundation
Identifies with/actively supports American Civil Liberties Union (ACLU)
Identifies with/actively supports Americans for Prosperity
Identifies with/actively supports MoveOn.org
Identifies with/actively supports National Association for the Advancement of Colored People (NAACP)
Identifies with/actively supports Red Cross
Identifies with/actively supports Chamber of Commerce
Identifies with/actively supports Alt Right
Identifies with/actively supports Freedom Caucus
Age
Education
Race/Ethnicity
Sex
Household Size
Housing Structure Type
Household Income
Marital Status
MSA status
Region (of 9)
Ownership Status of Living Quarters
Number Children 0-1yr
Number Children2-5yr
Number Children 6-12yr
Number Children 13-17yr
Employment Status

Table S4. Top 20 Covariates from Elastic Net Model Trained on All Covariates

1	Voted for whom in 2016 Presidential election
2	Housing structure type
3	Marital status
4	Identifies with/actively supports Alt Right
5	Identifies with/actively supports Freedom Caucus
6	Employment status
7	Identifies with/actively supports Indivisible
8	Identifies with/actively supports Chamber of Commerce
9	Household income
10	Ownership status of living quarters
11	Identifies with/actively supports Americans for Prosperity
12	Identifies with/actively supports Greenpeace
13	Shared opinion about town issue at public meeting in the past year
14	Could use common 60W light bulb in home
15	Contacted a government official in the past year
16	Political party
17	Volunteered/worked for political candidate/cause in the past year
18	Importance of acting patriotically in purchase decision
19	Partner's political identity
20	Identifies with/actively supports Amnesty International

The top 20 covariates are those that are shown to have the largest impact out of the full set of 63 covariates (shown in supporting information) in the elastic net model on consumer energy intensity choices.
